# Evaluation of ^134^Ce/^134^La-PSMA-617 for PET Imaging and Auger Electron Therapy of Prostate Cancer

**DOI:** 10.2967/jnumed.125.269751

**Published:** 2025-08

**Authors:** Kondapa Naidu Bobba, Anju Wadhwa, Anil P. Bidkar, Athira Raveendran, Luis Garcia, Nancy Greenland, David M. Wilson, Youngho Seo, Henry F. VanBrocklin, Robert R. Flavell

**Affiliations:** 1 Department of Radiology and Biomedical Imaging, University of California, San Francisco, California;; 2 Department of Nuclear Engineering, University of Tennessee-Knoxville, Knoxville, Tennessee;; 3 Department of Pathology, University of California, San Francisco, California;; 4 Helen Diller Family Comprehensive Cancer Center, University of California, San Francisco, California; and; 5 Department of Pharmaceutical Chemistry, University of California, San Francisco, California

**Keywords:** targeted radiopharmaceutical therapy, Auger electrons, [^134^Ce]Ce-PSMA-617, prostate cancer

## Abstract

The ^134^Ce/^134^La radionuclide pair has been proposed as a PET imaging surrogate for targeted α-radiotherapeutics. ^134^Ce decays to ^134^La via electron capture, emitting Auger electrons (AEs), which could be used for targeted radionuclide therapy. Additionally, the positron emission during this transition enables PET imaging, making ^134^Ce/^134^La a promising theranostic pair for prostate cancer. In this work, we investigated the potential of ^134^Ce for AE capture therapy using prostate-specific membrane antigen (PSMA)-617 for targeted radionuclide delivery. **Methods:** Radiolabeling of [^134^Ce]Ce-PSMA-617 proceeded as previously described, and C18 cartridge purification was optimized. In vitro, cell-binding and toxicity assays were performed on PSMA-positive PC3 PIP cells. In vivo PET imaging and ex vivo biodistribution studies were conducted on mice bearing dual PSMA-positive PC3 PIP and PSMA-negative PC3 flu tumor xenografts at various time points ranging from 1 to 72 h after injection. Additionally, an in vivo single-dose-treatment study was performed using 37- and 111-MBq doses in nude mice with PC3 PIP tumor xenografts. **Results:** PSMA-617 was successfully radiolabeled with ^134^Ce/^134^La and purified using the C18 cartridge method, achieving high molar activity (21.02 ± 0.11 MBq/nmol). Stability studies showed more than 95% stability in mouse serum at day 5. PSMA-positive PC3 PIP cells demonstrated 89.6% ± 0.55% cell binding, 55.45% ± 0.96% internalization at 24 h, and a dissociation constant of 32.9 ± 3.9 nM, comparable to other reported [^177^Lu]Lu/[^225^Ac]Ac-PSMA-617 radiocomplexes. In contrast, no cellular uptake or internalization was observed in PSMA-negative PC3 flu cells. Clonogenic assay of [^134^Ce]Ce-PSMA-617 showed a significant dose-dependent reduction in cell proliferation (*P* = 0.002). PET imaging revealed high tumor-specific uptake at early time points (1 and 4 h), followed by a gradual decline from 24 to 72 h, with rapid clearance from normal tissues. These results were corroborated by ex vivo biodistribution studies. In vivo therapy with [^134^Ce]Ce-PSMA-617 in tumor-bearing mice demonstrated a significant increase in median survival compared with control animals (saline, 33 d; 37 MBq, 50 d; and 111 MBq, 80 d, end of the study). **Conclusion:** [^134^Ce]Ce-PSMA-617 exhibited excellent in vitro and in vivo characteristics, providing significant survival benefits in mice. Collectively, these findings suggest that [^134^Ce]Ce-PSMA-617 is an effective theranostic agent for PET imaging and AE therapy of prostate cancer.

## Visual Abstract

**Figure:**
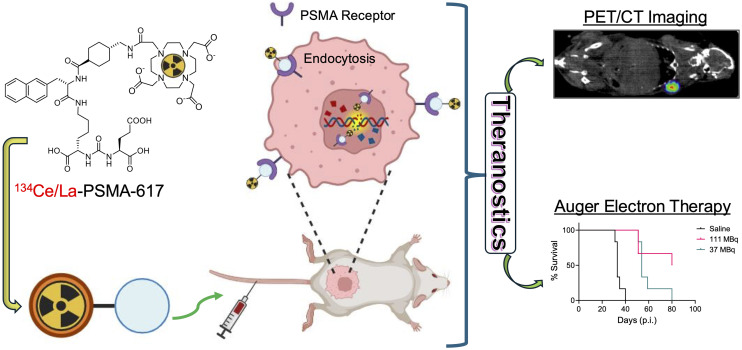

Radiopharmaceutical therapy has emerged as a promising approach for treating various malignancies, including prostate cancer, by utilizing specific targeting molecules and selecting appropriate radionuclides (1–3). High-energy β-particles have a long path length, thought to increase efficacy for bulky tumors (4). In contrast, Auger electrons (AEs) are emitted in a cascade, depositing energy over a short range (nanometers to micrometers), resulting in a high linear energy transfer (4–26 keV/μm) around the decay site (5). Although α- and β-targeted radiotherapies are well established, AE therapies have been less explored because of the requirement for close proximity to the target, limited availability of AE-emitting isotopes, and complex chemistry involved in their use. Despite these challenges, AE-emitting radionuclides, such as ^125^I, ^124^I, ^111^In, and ^58m^Co, have demonstrated effectiveness in targeted radionuclide therapy when delivered near the DNA of targeted cells, exhibiting lower toxicity than α- and β-therapy (6–8).

Prostate-specific membrane antigen (PSMA) has garnered significant interest as a therapeutic target in prostate cancer, especially following the approval of [^177^Lu]Lu-PSMA-617 (Pluvicto; Novartis) for the treatment of metastatic castration-resistant prostate cancer (9). PSMA is a type II membrane glycoprotein that is overexpressed in prostate cancer and further upregulated in metastatic disease (10). Radiopharmaceutical therapy utilizing [^177^Lu]Lu-PSMA-617 is effective, but responses are often not durable and therapy produces notable side effects, such as bone marrow suppression and xerostomia (11). Clinical trials are investigating [^225^Ac]Ac-PSMA-617 and other α-emitters, which show promising efficacy albeit at the potential increased risk of side effects, including xerostomia (12–14). There is an urgent need to develop or refine targeted treatments that maximize therapeutic efficacy while minimizing side effects in prostate cancer.

The radionuclide pair ^134^Ce/^134^La is a positron emitter with a half-life of approximately 3.2 d (Supplemental Fig. 1; supplemental materials are available at http://jnm.snmjournals.org), making it suitable for developing PET imaging applications, particularly when used with antibodies for late-time-point imaging (15–17). Recent studies have proposed that ^134^Ce/^134^La may serve as an imaging surrogate for targeted α-therapy with ^225^Ac (18). The decay of ^134^Ce to ^134^La emits strong characteristic AEs during the electron capture process (19). A prior study estimated the absorbed doses delivered to tumors and healthy tissues in theoretic models on both cellular and macroscopic levels, but no experimental evaluation of the therapeutic potential of ^134^Ce-based radiopharmaceuticals has been reported (19). PSMA-617 internalizes into prostate cancer cells via endocytosis, making it an intriguing carrier for AE-emitting radionuclides. Only a few AE-based PSMA-targeting agents, including [^58m^Co]Co‐PSMA‐617 and [^125^I]I-DCIBzL, were investigated for targeted radionuclide therapy, with promising effects and low toxicity in preclinical investigations (20).

In this study, we investigated for the first time (to our knowledge) the therapeutic potential of ^134^Ce AE therapy. ^134^Ce[Ce]-PSMA-617 was developed as a radiotheranostic agent, utilizing the therapeutic potential of AEs for therapy and positron emissions for simultaneous PET imaging.

## MATERIALS AND METHODS

### PET Imaging Methods

Small-animal PET/CT imaging was performed following a protocol similar to that reported in our previous publication (15). Mice received 4.8–5.0 MBq of ^134^Ce[Ce]-PSMA-617 for small-animal PET/CT imaging. For dynamic imaging, PET data were acquired in list mode and processed to produce a total of 42 dynamic frames (24 × 10 s, 12 × 30 s, and 6 × 330 s). The data were reconstructed using an iterative 2-dimensional ordered-subsets expectation maximization algorithm provided by the manufacturer, with both attenuation and scatter corrections applied. Images were normalized to the administered activity to parameterize images in terms of the percentage injected dose per milliliter (%ID/mL). Open-source AMIDE software was used to process the PET/CT images. PET images were acquired after CT acquisition for coregistration and attenuation correction.

### Ex Vivo Biodistribution Analysis

All mice in this study were euthanized via cervical dislocation under isoflurane anesthesia. Athymic male nude mice and PC3 PIP (left flank) and flu (right flank) tumor–bearing mice were euthanized at designated time points after injection of ^134^Ce[Ce]-PSMA-617 using a dose of 1.85 KBq. Blood was collected through cardiac puncture, and major organs including the liver, heart, kidney, lung, spleen, stomach, small intestine, large intestine, brain, muscle, bone, and tumors were collected, weighed, and analyzed in an automated γ-counter (Hidex) using energy windows of 450–580 KeV (for ^134^Ce). All the tissues were counted after at least 64.5 min of decay time (^134^La; half-life, 6.45 min) to reach secular equilibrium. Counts were decay-corrected to the time of injection, and the percentage injected dose per gram (%ID/g) was calculated by comparison with known radioactivity standards.

### In Vivo Therapy

Mice were randomized into 2 therapy and 1 control group, with 6 animals per group (PC3 PIP tumor mice only). The therapy groups received varying doses of [^134^Ce]Ce-PSMA-617 (37 and 111 MBq) in 0.9% sterile saline (∼100 μL) intravenously, whereas the control group received about 100 μL of saline. Tumor volumes were measured 3 times weekly until mice reached the endpoint criteria: decreased body condition score, severe petechiae, more than 20% weight loss, or tumor volume exceeding 2,000 mm³.

## RESULTS

### Radiolabeling of [^134^Ce]Ce-PSMA-617 and Stability Studies

**FIGURE 1.**
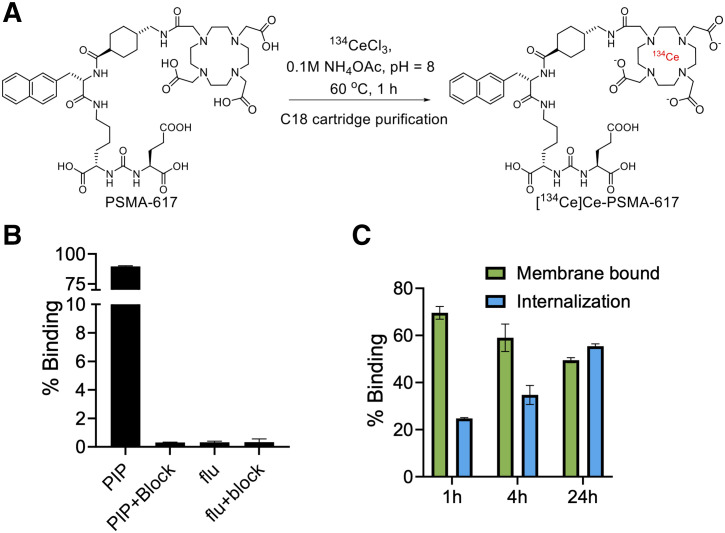
[^134^Ce]Ce-PSMA-617 can be efficiently prepared and demonstrates high cell binding and internalization in vitro. (A) Reaction scheme for radiolabeling and purification of [^134^Ce]Ce-PSMA-617. (B) Cell binding and blocking assay for [^134^Ce]Ce-PSMA-617 in PC3 PIP and flu cells (*n* = 3). High cell binding was observed in PSMA-positive PC3 PIP cells, whereas such binding was not seen in PSMA-negative PC3 flu cells (Supplemental Fig. 2). (C) Membrane-bound and internalization assay for [^134^Ce]Ce-PSMA-617 in PC3 PIP cells (*n* = 3) revealed rapid binding and internalization.

Radiolabeling of [^134^Ce]Ce-PSMA-617 (∼101.1 ± 0.11 MBq, *n* = 3) was performed in 95% ± 6.5% radiochemical yield using a commonly applied C18 cartridge purification method (21), with radiochemical purity above 99% and apparent molar radioactivity of 21.02 ± 0.11 MBq/nmol (*n* = 3) at the end of synthesis (non–decay-corrected; Fig. 1A). The stability of the [^134^Ce]Ce-PSMA-617 radiocomplex was assessed in mouse serum. No decomplexation was detected by radio–thin-layer chromatography after 5 d of incubation in the serum (Supplemental Fig. 2).

### In Vitro Cellular Uptake and Internalization Studies of [^134^Ce]Ce-PSMA-617

The cellular uptake of [^134^Ce]Ce-PSMA-617 was high for PSMA-positive PC3 PIP cells (89.6% ± 0.6%), whereas the PSMA-negative PC3 flu cells (0.31% ± 0.02%) showed negligible uptake. Moreover, the uptake in PC3 PIP cells was PSMA-specific since it could be blocked with an excess of 2-(phosphonomethyl)-pentanedioic acid (Fig. 1B). The degree of membrane binding versus internalization was evaluated in both PC3 PIP and PC3 flu cells. At 1 h, a high membrane-bound fraction of 69.6% ± 2.7% was observed, which decreased to 59.0% ± 5.8% and 49.5% ± 1.1% at 4 and 24 h, respectively. Internalized activity gradually increased over time, from 24.7% ± 0.4% to 34.8% ± 4.0% and 55.5% ± 1.0% at 1, 4, and 24 h, respectively (Fig. 1C). As expected, no evidence of specific internalization or membrane binding was seen in PC3 flu cells (Supplemental Fig. 3). The dissociation constant of the [^134^Ce]Ce-PSMA-617 was 32.9 ± 3.9 nM in PC3 PIP cells in a saturation binding assay. These results are similar to those for other therapeutic complexes, such as [^225^Ac]Ac/[^177^Lu]Lu-PSMA-617, reported in the literature (Supplemental Fig. 4) (21). Overall, the in vitro cell binding results demonstrated PSMA-targeted, efficient cell binding and rapid internalization of the [^134^Ce]Ce-PSMA-617 in PC3 PIP cells.

### In Vitro Cell-Killing Assays

**FIGURE 2.**
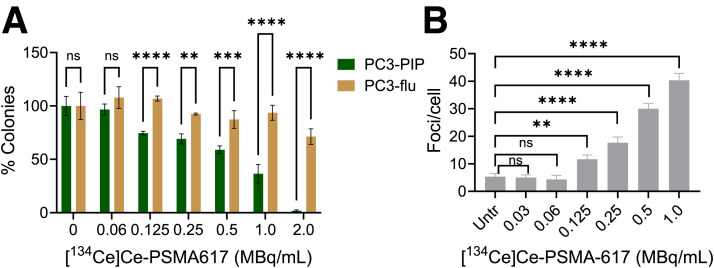
In vitro toxicity study for [^134^Ce]Ce-PSMA-617 in PC3 PIP cells. (A) Clonogenic survival assay for [^134^Ce]Ce-PSMA-617 showed dose-dependent decrease in colonies after treatment (*n* = 3). **P = 0.002. ***P = 0.0002. ****P < 0.0001. (B) DNA damage after treatment with various doses (0–1 MBq/mL) of [^134^Ce]Ce-PSMA-617 in PC3 PIP cells showing dose-dependent increase in DNA damage (*n* = 3). **P = 0.0036. ****P < 0.0001. ns = nonsignificant by Sidak multiple-comparison test; Untr = untreated.

A dose-dependent reduction in the number of colonies for PC3 PIP was observed in a colony-forming assay (Fig. 2A; Supplemental Fig. 5). A range of 96.7% ± 5.1% to 1.8% ± 0.9% viable was observed after treatment with 0.06–2 MBq mL^−1^, with a calculated half-maximal effective concentration of 1.5 ± 0.1 MBq/mL (Supplemental Fig. 6). In contrast, this effect was not seen on PC3 flu cells. A dose-dependent increase in γH2AX foci, indicating DNA damage, was observed afterward in [^134^Ce]Ce-PSMA-617–treated PC3 PIP cells (Fig. 2B; Supplemental Fig. 7). At higher doses of 2–4 MBq/mL, foci could not be counted because of cell death and nuclear fragmentation (Supplemental Fig. 8). Taken together, these results demonstrate that [^134^Ce]Ce-PSMA-617 has substantial PSMA-dependent therapeutic efficacy in vitro.

### Small-Animal PET/CT Imaging and Ex Vivo Biodistribution

**FIGURE 3.**
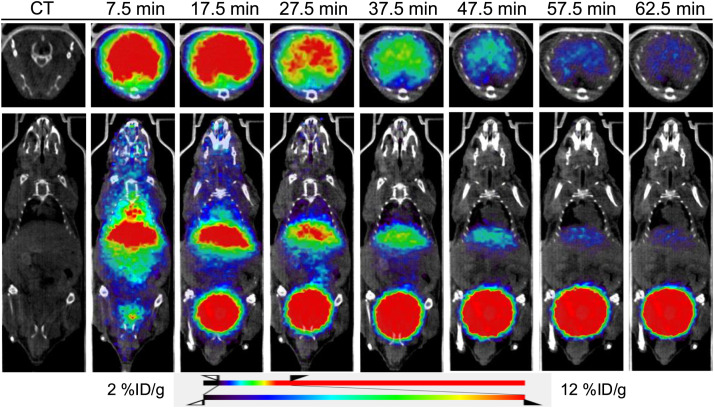
Dynamic coronal fused small-animal PET/CT images obtained after injection of [^134^Ce]Ce-PSMA-617 in PC3 PIP (left shoulder) and PC3 flu (right shoulder) tumor xenografts in athymic nude mice (*n* = 3). High liver uptake was observed at early time points because of free ^134^La caused by nuclear recoil effect. This uptake gradually decreased over time, showing minimal or no liver uptake at later time points because of short half-life of ^134^La (∼6.45 min).

Dynamic PET imaging was performed after [^134^Ce]Ce-PSMA-617 administration in nude mice bearing PC3 PIP (left shoulder) and PC3 flu (right shoulder) tumor xenografts over 1 h (*n* = 4). Figure 3 shows that the high accumulation of the tracer in the liver at initial time points gradually decreased over the 1-h imaging period (Fig. 3). This decrease likely reflects the accumulation of free ^134^La due to the nuclear recoil effect, both because of the presence of ^134^La in the injected formulation of [^134^Ce]Ce-PSMA-617 and because of in situ generation from [^134^Ce]Ce-PSMA-617 in vivo during the blood circulation, as reported previously (15). A gradual increase in the bladder was observed, consistent with renal clearance and excretion of the tracer.

**FIGURE 4.**
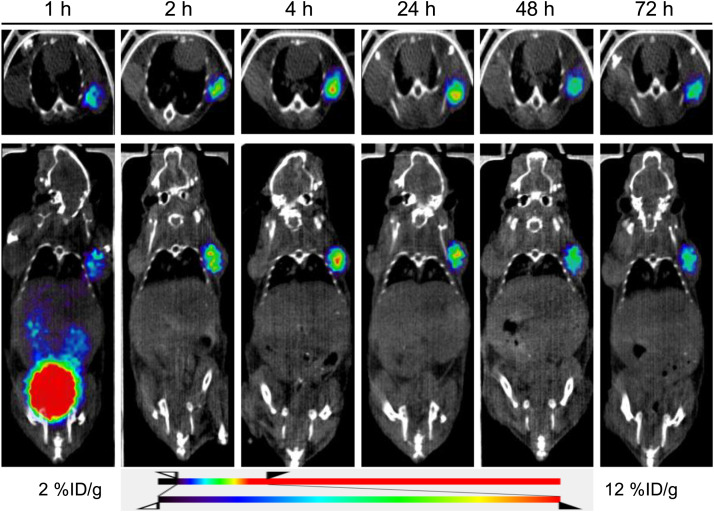
Transverse and coronal PET/CT images obtained up to 72 h after [^134^Ce]Ce-PSMA-617 injection in mouse bearing PSMA-positive PC3 PIP (left shoulder) and PSMA-negative PC3 flu (right shoulder) tumor xenografts, demonstrating gradual increase in tumor uptake up to 4 h and slow decline from 24 to 72 h (*n* = 4).

Multiple-time-point static scans were acquired until 72 h. High tumor uptake was observed at 4 h after injection; 5.64 ± 1.86 %ID/g was found through the region of interest, gradually declining until up to 72 h (3.64 ± 1.38 %ID/g). No uptake was observed in the PC3 flu tumors (Fig. 4; Supplemental Fig. 9). The uptake in other significant organs was also investigated using region-of-interest analysis (Supplemental Table 1). The %ID/g values at 1, 2, 4, 24, 48, and 72 h were, respectively, 1.38 ± 0.51, 0.21 ± 0.08, 0.16 ± 0.09, 0.08 ± 0.06, 0.07 ± 0.07, and 0.07 ± 0.05 for blood; 2.98 ± 0.75, 0.46 ± 0.14, 0.35 ± 0.13, 0.22 ± 0.12, 0.18 ± 0.13, and 0.17 ± 0.08 for liver; and 11.07 ± 3.60, 2.66 ± 0.86, 1.72 ± 0.59, 0.47 ± 0.30, 0.31 ± 0.24, and 0.21 ± 0.17 for kidney. A high initial uptake at 1 h was observed in these organs, with most of the activity gradually clearing over time, starting at 2 h and continuing to 72 h.

**FIGURE 5.**
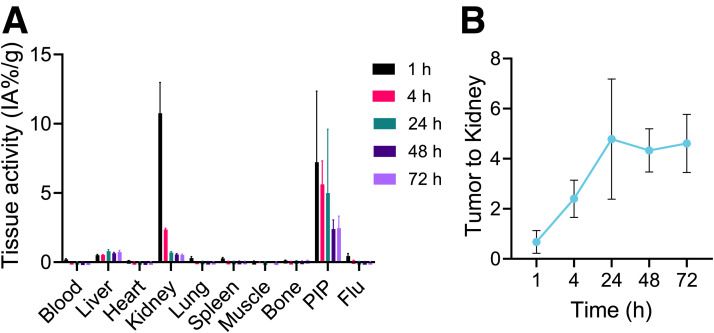
Multiple-time-point ex vivo biodistribution analysis. (A) Ex vivo biodistribution of [^134^Ce]Ce-PSMA-617 in mouse bearing PSMA-positive PC3 PIP (left shoulder) and PSMA-negative PC3 flu (right shoulder) tumor xenografts until 72 h after injection (*n* = 4). (B) Tumor-to-kidney ratios derived from ex vivo biodistribution analysis.

A separate ex vivo biodistribution study was performed on dual PC3 PIP and PC3 flu tumor xenografts (Fig. 5A; Supplemental Table 2). The %ID/g values were 7.22 ± 5.13, 5.62 ± 1.70, 4.99 ± 4.61, 2.39 ± 0.66, and 2.45 ± 0.87 for tumor and 10.76 ± 2.21, 2.35 ± 0.11, 0.7 ± 0.06, 0.55 ± 0.08, and 0.52 ± 0.01 for kidney at 1, 4, 24, 48, and 72 h, respectively. As expected, most of the [^134^Ce]Ce-PSMA-617 cleared from the other nontargeted organs over time. High tumor-to-background ratios were observed (Supplemental Table 3). The tumor-to-kidney ratio increased from 0.68 ± 0.45 %ID/g at 1 h after injection to 4.61 ± 1.16 %ID/g at 72 h after injection (Fig. 5B). Tumor-to-muscle ratios increased from 116.3 ± 37.8 %ID/g at 1 h after injection to 475.3 ± 146.8 %ID/g at 4 h after injection and later slowly dropped to 204.7 ± 119.8 %ID/g at 72 h after injection (Supplemental Fig. 10A). Tumor-to-blood ratios increased over time from 40.34 ± 32.3 %ID/g at 1 h after injection to 122.8 ± 36.9 %ID/g at 72 h after injection (Supplemental Fig. 10B). The tumor-to-liver ratios continuously dropped from 14.4 ± 10.4 %ID/g at 1 h after injection to 3.3 ± 1.1 %ID/g at 72 h after injection (Supplemental Fig. 10C).

### Tumor and Whole-Body Dosimetry

On the basis of prior publications evaluating AE electron therapy (3,8), we selected administered activities of 37 and 111 MBq for treatment studies. To corroborate these choices, we performed radiation dosimetry for the tumors from the ^134^La PET imaging data that were acquired at multiple time points. With the assumption that the tumor geometry is a sphere, we estimated the absorbed doses in tumors, incorporating dose contributions from the AEs of ^134^Ce decays as well as the γ-rays and positrons of ^134^La decays. From this estimation, we found that 16.8 mGy/MBq (8.2 and 8.5 mGy/MBq from ^134^Ce and ^134^La, respectively) was the average absorbed dose expected for the therapy experiments. For 37 MBq, the expected absorbed doses are 0.30 and 0.32 Gy from ^134^Ce and ^134^La, respectively, and for 111 MBq, they are 0.91 and 0.95 Gy, respectively. Organ and effective whole-body doses were calculated and are reported in Supplemental Table 4. The urinary bladder received the highest dose, 62.1 ± 23.6 × 10^−^³ mSv/MBq, indicating it as the dose-limiting organ. The liver (5.3 ± 1.0 × 10^−^³ mSv/MBq) and kidneys (4.2 ± 2.2 × 10^−^³ mSv/MBq) had comparable doses, whereas all other tissues received less than 2.2 × 10^−^³ mSv/MBq. The whole-body effective dose was 3.8 ± 1.2 × 10^−^³ mSv/MBq.

### Therapeutic Potential of [^134^Ce]Ce-PSMA-617 in PC3 PIP Prostate Cancer Xenografts

**FIGURE 6.**
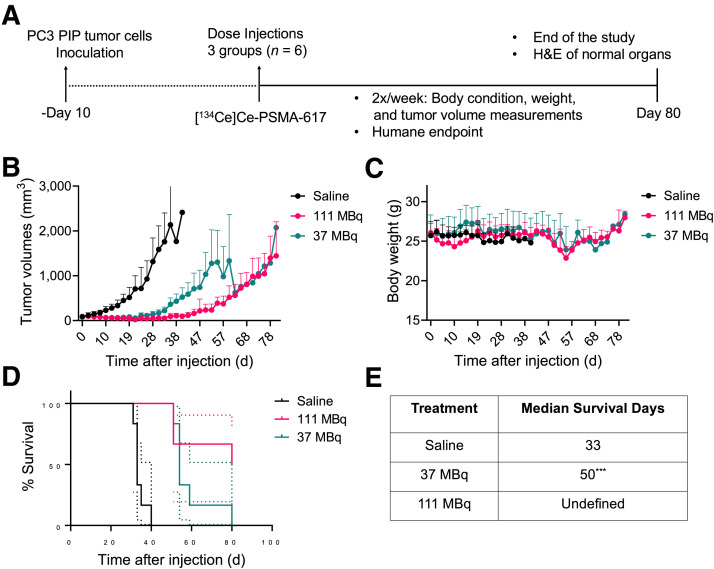
Single-dose-treatment study of [^134^Ce]Ce-PSMA-617 in PC3 PIP tumor xenografts. (A) Schematic illustration for single-dose-treatment study of [^134^Ce]Ce-PSMA-617 with 37-MBq dose, 111-MBq dose, and saline as control. (B) Average tumor volumes. (C) Body weight. (D) Kaplan–Meier plot for treatment and vehicle cohorts. Dotted lines indicate 95% CI for survival probability. (E) Treatment group’s median survival days and statistical analysis (*n* = 6). High dose (111 MBq) showed prolonged survival, and 37 MBq showed significant difference vs. control saline group. ***P = 0.0005. H&E = hematoxylin and eosin; Undefined = not reached endpoint.

The therapeutic potential for ^134^Ce-DOTA-PSMA-617 was assessed in PC3 PIP xenografts. Mice were randomized into 3 groups (6 mice/group) (Fig. 6A) receiving 37 or 111 MBq of [^134^Ce]Ce-PSMA-617 or saline vehicle. The [^134^Ce]Ce-PSMA-617 groups demonstrated improvement in tumor volumes and overall survival (Fig. 6B), and the group receiving the higher dose, 111 MBq, showed prolonged survival in comparison with the 37-MBq and saline control groups. The saline and 37-MBq groups showed consistent body weights. However, a transient reduction of up to 7.6% in body weight was observed in mice treated with 111 MBq of [^134^Ce]Ce-PSMA-617 at day 10, which recovered by day 19 (Fig. 6C). Median survival at the humane endpoint was 33 d for the saline control group and 50 d for the 37-MBq group—a significant difference (*P* < 0.0001). In contrast, the higher dose (111 MBq) showed a prolonged, significant tumor growth inhibition, with half the mice still alive at day 80. One mouse reached the tumor volume humane endpoint on day 80, and 2 mice were euthanized on day 54 because of delayed body weight reduction (∼20%) (Fig. 6D). Histologic evaluation of organs including heart, lung, bone marrow, spleen, liver, and kidney was normal (Supplemental Fig. 11) except for mild chronic centrizonal inflammation in the liver of one of the 111-MBq mice, aggregates of macrophages in the lungs of one of the 111-MBq mice (Supplemental Fig. 12), and tumor in the lungs of one of the saline control mice (Supplemental Fig. 13).

## DISCUSSION

Targeted therapy using AE emitters has demonstrated promise in preclinical studies; however, clinical applications remain limited, with no agents currently approved for use (5). The first agent tested for targeted AE therapy was the thymidine analog ^125^I-5-iodo-2′-deoxyuridine, used in a murine early ascites tumor model for ovarian cancer (22). Subsequent evaluations in other cancer models revealed greater efficacy but also unwanted toxicity, primarily because of the in vivo instability of the tracer (23).

After the success of PSMA-targeted radiopharmaceuticals for prostate cancer imaging and therapy, the Auger emitter ^125^I was used to develop [^125^I]I-DCIBzL (20), which showed excellent antitumor efficacy in prostate cancer therapy compared with phosphate-buffered saline controls. Other PSMA-targeted agents, including [^58m^Co]Co-PSMA-617 (24), also demonstrated promising preclinical antitumor efficacy. Additionally, antibody-based agents such as [^111^In]In-NLS-trastuzumab for breast cancer (25), [^111^In]In-anti-CD74 for B-cell lymphoma (26), and [^111^In]In-J591 (27) for prostate cancer showed strong therapeutic efficacy in preclinical studies. However, none of these agents have been translated into clinical practice.

In 2002, Lubberink et al. proposed the ^134^Ce/^134^La theranostic matched pair for radionuclide therapy applications, using theoretic models to estimate Auger emissions for single-cell and whole-body dosimetry. They reported that microscopic tumor-absorbed doses from ^134^Ce/^134^La (12.4 mGy/MBq) were higher than those from ^111^In (0.98 mGy/MBq) and ^131^I (8.17 mGy/MBq) and comparable to those from ^90^Y (12.1 mGy/MBq) (19). Despite its potential, ^134^Ce/^134^La has not been evaluated for therapeutic applications. Recent advancements in the production of ^134^Ce/^134^La by the Department of Energy have facilitated studies of its PET imaging properties, positioning it as a promising imaging surrogate for targeted α-therapies (15,16,18,28).

[^134^Ce]Ce-PSMA-617 exhibited excellent cell binding (89.6% ± 0.55%) and high specific internalization (55.45% ± 0.96%) in PC3 PIP cells at 24 h, compared with PC3 flu cells. These results align with findings for other PSMA-targeting agents such as [^125^I]I-DCIBzL (20) and [^58m^Co]Co-PSMA-617 (24). The saturation binding constant of [^134^Ce]Ce-PSMA-617 (32.9 ± 3.9 nM) was similar to those of established agents, such as [^177^Lu]Lu/[^225^Ac]Ac-PSMA-617 agents, in PC3 PIP cells (21).

In vivo PET imaging of [^134^Ce]Ce-PSMA-617 confirmed high tumor uptake in PC3 PIP tumors compared with PC3 flu tumors. Ex vivo biodistribution studies corroborated this finding, including region-of-interest analysis, except at the early time point of 1 h after injection, at which we observed differences in tumor uptake. These can be attributed to the redistribution of ^134^La due to the nuclear recoil effect, as described in a recent publication (28). Interestingly, whereas other Auger PSMA agents, such as ^55^Co-DOTA-PSMA-617 (imaging surrogate for [^58m^Co]Co-PSMA-617) (24) and [^125^I]I-DCIBzL (20), showed high uptake (>50 %ID/g) at 1 h after injection, [^134^Ce]Ce-PSMA-617 showed only 12 %ID/g, possibly because of the influence of the ^134^Ce/^134^La radiometal. Importantly, the reduced kidney uptake of [^134^Ce]Ce-PSMA-617 may mitigate nephrotoxicity, a challenge in PSMA-based therapies.

A single-dose-treatment study was conducted with 37- and 111-MBq activity levels in PC3 PIP tumors. The selection of these activity levels was based on 2 considerations. The first is that Lubberink et al. (19) estimated microscopic tumor-absorbed doses to be higher than those of ^111^In (discussed above), and the second is that other PSMA-targeted Auger agents, such as [^125^I]I-DCIBzL and [^58m^Co]Co-PSMA-617, demonstrated minimal toxicity and extended antitumor efficacy in tumors at a similar level. A single dose of [^134^Ce]Ce-PSMA-617 (37 MBq) showed significant tumor growth inhibition compared with control groups (*P* = 0.0005), and the higher dose (111 MBq) showed prolonged survival, with half the mice still alive at day 80. These findings are consistent with studies by Shen et al. (9), who observed therapeutic effects of [^125^I]I-DCIBzL at doses ranging from 0.37 to 111 MBq. In contrast, [^58m^Co]Co-PSMA-617 (144 ± 9 MBq) studies reported shorter survival times (23 d) under similar conditions, possibly because of differing study endpoints (24).

From the tumor dosimetry, the expected absorbed doses are 0.30 and 0.32 Gy for 37 MBq and 0.91 and 0.95 Gy for 111 MBq from ^134^Ce and ^134^La, respectively. What this absorbed dose estimation indicates is that, without significantly elevated relative biological effectiveness, the therapeutic effect shown in Figure 6 is not well explained. For example, in external-beam radiation therapy settings, even 20 Gy of x-irradiation have shown minimal effects on suppression of tumor growth (29). These conflicting data suggest a need for further studies to investigate the therapeutic efficacy, toxicity, and dosimetry of AE therapies such as [^134^Ce]Ce-PSMA-617.

Although some body weight loss indicating toxicity was observed at the highest dose level tested, histopathologic analysis in our study showed no significant damage in normal tissues, including bone marrow, between treated and untreated mice. This observation aligns with the radiobiologic properties of auger emitters, which demonstrate increased efficacy when internalized into cells (30).

This study had several limitations, which motivate areas for future investigation. First, the study did not establish the maximum tolerated dose. Acute and chronic toxicity studies, along with blood chemistry profiles, are needed for a comprehensive assessment. Second, fractionated regimens were not investigated, which could improve long-term outcomes and manage potential toxicity. A final limitation is that high-purity ^134^Ce/^134^La is essential for the clinical translation of this agent, as the current production includes ^139^Ce (half-life, 137.6 d) as an impurity. Because of its long half-life, ^139^Ce may cause difficulties for patients. For example, since the PSMA agents wash out rapidly, waste management could be challenging, highlighting the need for improved production and purification methods.

## CONCLUSION

This study demonstrates the promising potential of [^134^Ce]Ce-PSMA-617 as a theranostic agent for targeted AE therapy in prostate cancer. Our results indicate high tumor specificity, limited off-target effects, and significant tumor growth inhibition with prolonged survival in preclinical models. The minimal uptake in dose-limiting organs, particularly kidneys and salivary glands, highlights its safety profile, whereas its limited crossfire effects suggest its suitability for treating small tumors or metastatic disease. Further studies evaluating long-term toxicity, fractionated dosing, and DNA damage mechanisms are warranted to realize its clinical potential fully.

## DISCLOSURE

The ^134^Ce used in this research was supplied by the U.S. Department of Energy Isotope Program, managed by the Office of Isotope R&D and Production. This study was supported by U.S. Department of Energy grant DE-SC0023467 and NIH R01 CA279203. No other potential conflict of interest relevant to this article was reported.

## Notes

KEY POINTS **QUESTION:** Can the AEs from the ^134^Ce/^134^La decay scheme be utilized for AE capture therapy applications? **PERTINENT FINDINGS:** An AE capture–based theranostic agent, [^134^Ce]Ce-PSMA-617, demonstrated high tumor specificity, minimal off-target effects, and significant tumor growth inhibition, leading to prolonged survival in preclinical prostate cancer models. The low uptake in dose-limiting organs supports its favorable safety profile, whereas limited crossfire effects suggest suitability for small tumors or metastatic disease. **IMPLICATIONS FOR PATIENT CARE:** The preliminary results highlight the strong potential of [^134^Ce]Ce-PSMA-617 for clinical translation to mitigate the challenges associated with [^177^Lu]Lu-PSMA-617 treatments in the clinic.
